# Collagenous gastritis: clinical features, histologic correlates and unanswered questions

**DOI:** 10.1111/his.15542

**Published:** 2025-09-05

**Authors:** Jiannan Li, Kevin Tanager, Namrata Setia

**Affiliations:** ^1^ Department of Pathology and Immunology Washington University School of Medicine St. Louis Missouri USA; ^2^ Department of Pathology University of Chicago Medicine Chicago Illinois USA

**Keywords:** atrophic gastritis, celiac, chronic anaemia, collagenosis, collagenous gastritis, gastric nodularity, *Helicobacter pylori*, lymphocytic gastritis

## Abstract

Collagenous gastritis (CG) is a rare gastrointestinal disorder characterized by subepithelial collagen deposition and lamina propria inflammation. Despite its first description over four decades ago, the pathogenesis remains unclear, with no standardized pathologic criteria/classification, treatment or established prognosis. A systematic PubMed search identified all English‐language case reports, series and observational studies describing CG. Data on demographics, clinical presentation, endoscopic appearance and histologic features were extracted. Of the 133 patients with available demographic data, 101 patients had corresponding histologic information, reflecting the overall rarity and limited characterization of collagenous gastritis in the literature. The most common presenting symptoms were abdominal pain and chronic anaemia. A subset of patients had concurrent collagenous colitis, collagenous sprue, *Helicobacter pylori* gastritis or celiac disease. The predominant endoscopic finding was gastric mucosal nodularity, typically diffuse or corpus‐predominant. Collagen band thickness ranged widely, with a median of 50.5 μm and a maximum of 225 μm. Band distribution was most commonly pan‐gastric or corpus‐predominant. Among the proposed histologic subtypes of CG, the atrophic pattern was most frequently observed and often correlated with isolated gastric involvement and lack of clinical or histologic remission. This review highlights that histologic classification may help guide differential diagnoses and prognosis. Accordingly, we advocate for explicit reporting of these features in pathology reports. Key gaps in pathogenesis, including the roles of environmental and genetic factors, are also reviewed. This review synthesizes current knowledge of CG and underscores the need for further studies to clarify disease mechanisms. Improved histologic classification and exploration of underlying aetiologies may enhance diagnosis, treatment and research in this underrecognized condition.

## Graphical abstract

This study provides a comprehensive overview of collagenous gastritis, offering pathologists an updated perspective that is valuable for clinical practice and guiding future research, as well as supporting the development of standardized reporting procedures.

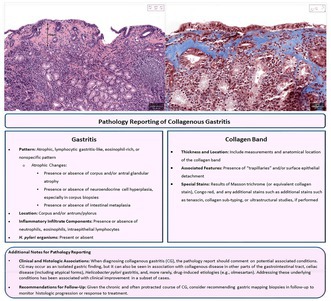

**Abbreviations**
AIG: autoimmune gastritis
CC: collagenous colitis
CG: collagenous gastritis
CGE: collagenous gastroenteritis
CT: computed tomography
CVID: common variable immune deficiency
ECM: extracellular matrix
H&E: hematoxylin and eosin
*H. pylori*
: *Helicobacter pylori*
HIV: Human Immunodeficiency Virus
MRI: magnetic resonance imaging

## Introduction

Collagenous gastritis (CG) is a rare disorder characterized by prominent subepithelial collagen deposition, usually exceeding 10 μm, in association with inflammatory changes in the gastric mucosa. It was first described in 1989,^1^ and its prevalence is estimated to be 13 per 100,000 upper gastrointestinal endoscopies.^2^ CG is considered part of the broader spectrum of collagenous gastroenteropathies, which includes conditions like collagenous sprue and collagenous colitis.^3^, ^4^ The literature typically categorizes CG into paediatric and adult forms based on the age of presentation. Despite its initial description over four decades ago, the pathogenesis of CG remains incompletely understood. Standardized pathological criteria, classification systems and evidence‐based treatment guidelines are lacking, and the overall prognosis remains uncertain. This comprehensive review aims to provide a clearer understanding of CG by synthesizing the available clinical and pathological data, while also highlighting the existing knowledge gaps in the literature.

## Methods

A PubMed literature search was conducted using the term ‘collagenous gastritis’, limited to English‐language publications. We review and summarize the available literature, including the references listed below and supplemental references not cited in the main text, to evaluate the clinical and histologic characteristics of CG. Clinical data including demographics, presentation and endoscopic and imaging findings are summarized. Histologic features were assessed with a focus on the main diagnostic components: the collagen band and the inflammatory changes in the gastric mucosa. Based on the extracted information, we propose key histologic features to be included in pathology reports and highlight unanswered questions related to the pathogenesis of CG. Ethics approval and/or informed consent were not required for this study. The data that support the findings of this study are available from the corresponding author upon reasonable request.

## Clinical Features

### Clinical Presentation

Collagenous gastritis affects individuals across a broad age range but demonstrates a predilection for younger patients. Of the 133 patients with available demographic information, the reported median age at diagnosis is 18 years, with the majority of cases occurring in individuals younger than 40 years (±20.66). A female predominance is also observed, accounting for approximately two‐thirds of the cases described in the literature.

Most patients with CG present with at least one symptom; only five patients in the reviewed literature were reported as asymptomatic.^5^, ^6^, ^7^, ^8^ The nature and severity of presenting symptoms vary widely. The most commonly reported initial manifestations include abdominal pain and chronic anaemia, each occurring in nearly half of symptomatic cases. Abdominal pain ranged from mild, cramp‐like discomfort, sometimes postprandial or bloating‐related, to severe, recurrent or progressively worsening episodes. Chronic anaemia also presented with variable severity, from incidental laboratory findings to more overt signs such as pallor, exercise intolerance, palpitations, pica and resistance to iron therapy. Anaemia is likely secondary to chronic blood loss from entrapped dilated capillaries, potentially leading to iron deficiency.^9^ Concurrent gastric hypochlorhydria may further contribute to iron deficiency by impairing iron absorption.^10^

Less frequently reported symptoms include nausea, early satiety, vomiting and weight loss as well as altered bowel habits, most commonly diarrhoea and, less frequently, constipation. Rare reports also describe dysphagia.^11^ The literature also includes reports of severe clinical manifestations of CG, including profound weight loss,^12^, ^13^ gastric perforation^14^, ^15^ and generalized body swelling.^16^ Given the broad spectrum of nonspecific symptoms, many of which overlap with more common gastrointestinal conditions, CG is rarely considered high on the clinical differential diagnosis, particularly in patients without associated systemic or autoimmune conditions.

Collagenous gastritis may occur either in isolation or in conjunction with other gastrointestinal disorders, with approximately half of CG cases being isolated. A key association with CG is a collagenosis at other sites in the gastrointestinal tract, namely collagenous colitis (CC) and collagenous sprue. When either or both of these co‐occur with CG, it can be conceptualized as collagenous gastroenteritis (CGE). In the reviewed cases, collagenous colitis was observed in nearly 25% of CG patients, while collagenous sprue co‐occurred in approximately 10%. Notably, eight patients exhibited involvement of the stomach, small intestine and colon, reflecting pan‐gastrointestinal involvement in CGE.^15^ Of note, isolated collagenous gastritis is more commonly found in young patients,^17^, ^18^, ^19^ with an increased prevalence in children.^10^ Another potential association is with *Helicobacter pylori* gastritis, which was reported in about 10% of CG cases.^10^ In several of these cases, *H. pylori* infection preceded the development of CG and treatment of the infection led to partial or complete resolution of CG features, suggesting a potential pathophysiological connection.^20^ Also, celiac disease and celiac‐like duodenal changes show an apparent association, co‐occurring in about a tenth of CG patients, with variable histopathologic severity and gluten‐avoidance responsiveness.^15^

Collagenous gastritis has been identified in patients with a variety of autoimmune disorders, including type I diabetes,^21^ autoimmune hypothyroidism,^22^ psoriasis,^21^ systemic lupus erythematosus, autoimmune haemolytic anaemia,^23^ Sjögren syndrome,^24^ Hashimoto's thyroiditis^15^ and Behçet's disease.^25^ Notably, several cases have been associated with immune deficiencies, including primary selective IgM deficiency,^26^ hypogammaglobulinemia,^27^ common variable immune deficiency (CVID)^28^ and HIV infection.^29^ Although rarely reported, drug‐induced CG, specifically linked to olmesartan, underscores the importance of considering medication‐related causes, particularly in older individuals.^18^, ^25^, ^30^ While the pathogenesis of CG remains unclear, its associations with collagenous gastroenteritis, *H. pylori* gastritis, celiac sprue, autoimmune disorders and immune deficiencies provide potential avenues for further investigation into common underlying pathobiology.

### Endoscopy Findings

**Figure 1:**
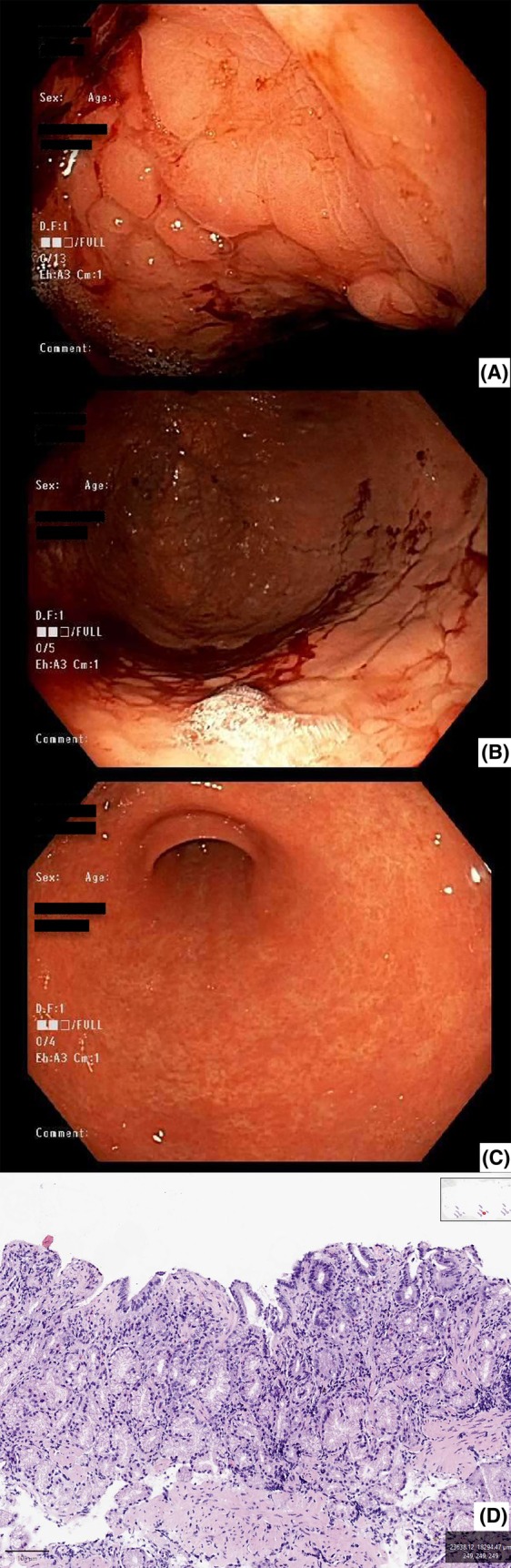
A 60‐year‐old underwent esophagogastroduodenoscopy for evaluation of abdominal pain. Endoscopy revealed diffusely nodular lesions in gastric body/fundic mucosa (**A**), associated with mucosal friability and haemorrhage (**B**) and sparing of antral mucosa (**C**). Histologic evaluation demonstrated an atrophic pattern of collagenous gastritis in the body/fundic mucosa (**D**, H&E, 100×).

Similar to the majority of CG patients experiencing at least one symptom, most patients also show some form of gastric abnormality on endoscopy, with only 10% showing normal endoscopy.^8^, ^16^, ^21^, ^23^, ^25^, ^31^, ^32^, ^33^, ^34^ The most common endoscopic finding is gastric mucosal nodularity, seen in more than half of patients, with a significant minority by contrast showing mucosal atrophy. Descriptions of the nodularity by endoscopists can vary, with appearances resembling polypoid lesions, prominent rugae or oedematous mucosa.^7^, ^35^, ^36^ Less common findings include friability, erythema, erosion and ulceration, scarring and, in rare cases, haemorrhage.^37^ A representative case demonstrating these findings is shown in Figure 1. Although nodularity/polypoid mucosa are endoscopic abnormalities that warrant biopsy and microscopic examination, it is the biopsy of the area in between the nodules that has a higher diagnostic yield, as the collagen bands frequently only appear in between the nodules.^12^

Among the subset of reviewed cases with delineated endoscopic findings, the abnormalities are most commonly observed diffusely across the stomach, followed by a gastric corpus‐predominant pattern and then an antrum‐predominant pattern. In one cohort of 40 patients, 71% of patients exhibited a corpus‐predominant involvement and 12.5% antrum‐predominant involvement.^38^ Another large cohort of 31 patients reported 70% of patients having both corpus and antrum involvement.^18^ Some CG patients showed unrelated endoscopic findings such as fundic gland polyps and hiatal hernia.^39^ Beyond the stomach, the duodenum is frequently involved endoscopically in CG, often presenting as nodularity^21^, ^40^ or inflammation.^25^, ^41^

## Imaging

Imaging studies are not commonly used in the diagnosis of CG. Among reviewed cases, fewer than a quarter of patients underwent some form of imaging, generally as part of a broader evaluation for generalized abdominal symptoms. Accordingly, conventional imaging modalities such as abdominal CT and X‐ray were most often employed to assess abdominal pain, diarrhoea or constipation, or constitutional symptoms including weight loss and failure to thrive. In one patient, an abdominal X‐ray performed for acute abdominal pain revealed subdiaphragmatic free air, suggestive of gastrointestinal perforation.^15^ Other imaging techniques including barium radiography, CT enterography and MRI, by contrast, were performed in a smaller subset of patients, often as part of a targeted gastrointestinal workup, for symptoms such as dysphagia, or in the context of gastric cancer surveillance.^11^, ^24^

When performed, barium radiography and endoscopic ultrasound were the most likely to demonstrate abnormalities attributable to CG. Barium studies may show a mosaic‐like gastric mucosal pattern, while ultrasonography may reveal gastric mucosal thickening, findings that could be contributory to the final diagnosis.^42^, ^43^ However, neither barium studies nor endoscopic ultrasound are sufficiently sensitive or specific to reliably exclude CG.^24^, ^44^, ^45^ CT and MRI demonstrate even lower diagnostic sensitivity and specificity, rarely identifying abnormalities attributable to CG when performed.^25^, ^46^, ^47^, ^48^

## Histopathologic Features

Histologic data were available for 101 patients included in our review. The pathologic injury in CG involves two diagnostic features: the abnormal collagen band and the inflammatory changes in the gastric mucosa. In the following sections, each of these components are discussed based on the review of literature with a goal to better characterize the disease and understand their roles in the pathogenesis.

### Abnormal Collagen Band

The diagnostic criteria for CG require the subepithelial collagen thickness to be greater than 10 μm. The median collagen thickness reported in the literature is 50.5 μm, with cases showing bands as thick as 225 μm.^49^ The distribution of the collagen band varied, with most cases demonstrating thickening at all sites or gastric corpus only, in roughly equal proportions. Involvement limited to the antrum is relatively uncommon and may be an artefact of incomplete sampling, specifically due to the corpus mucosa not being biopsied for histologic evaluation.^15^, ^40^, ^50^

The collagen band in the majority of reported cases and case series tends to be irregular and focal, often exhibiting a ragged pattern along the lower edge that entraps lamina propria cellular elements and small capillaries, also referred to as ‘trapillaries’.^9^, ^51^, ^52^, ^53^ In most cases, the collagen band does not extend into the deeper portions of the lamina propria.^1^ Additionally, the collagen band is frequently associated with damage and injury to the surface epithelium, often described as flaking, stripping, denudation, detachment or disruption.^9^, ^53^, ^54^, ^55^, ^56^, ^57^

In some reported cases, the collagen band and the diagnosis of CG were established after multiple repeat endoscopies. These cases typically presented initially with chronic active gastritis, followed by focal fibrosis and ultimately the formation of a collagen band thicker than 10 μm, meeting the diagnostic criteria for CG.^5^, ^20^

Conventionally, Masson trichrome and Congo red stains are performed in suspected cases to confirm and assess the thickness of collagen bands, and exclude the presence of amyloid deposition, respectively. Ultrastructural examination has been performed in a few cases and reports a patchy subepithelial band of haphazardly arranged collagen fibres with entrapped capillaries, lymphocytes, eosinophils, mast cells and fibroblasts. The overlying surface epithelium shows a focal decrease in mucous granules, widening of intercellular spaces and detachment from the basement membrane in some areas. Fibroblasts entrapped in the subepithelial collagen bundles and located around foveolae and glands do not show significant alterations. Many of the entrapped eosinophils and mast cells show evidence of prominent degranulation. Additionally, focal thickening of the basement membrane of the atrophied glands and the blood vessels is also reported.^49^, ^58^

Collagen typing has been evaluated in several studies, all consistently showing negative staining for collagen type IV—a network‐forming collagen that is a key structural component of the basement membrane in the gastrointestinal tract.^8^, ^34^, ^59^ This absence of collagen IV staining is observed in both CG and CC cases. By contrast, staining for interstitial collagens, type I and III—fibrillar‐type collagens located beneath the basement membrane, forming the main structural framework of the lamina propria and submucosa—shows different patterns in CG and CC. In CC, the abnormal subepithelial collagen band strongly stains for collagen types I and III, and also for collagen type VI.^60^, ^61^ Similar studies in CG are sparse but report variable positivity for collagen types III and VI.^62^ It is important to note that Masson trichrome, the most commonly used histochemical stain to highlight collagen, detects all collagen types, including I, III, IV and VI.^63^

Among components of the extracellular matrix, tenascin has emerged as the most extensively studied and promising marker in CG, with tenascin positivity consistently reported across all CG cases. Tenascin is a large extracellular glycoprotein, most often expressed in response to tissue injury and is considered a marker of active tissue remodelling with high turnover. Tenascin‐rich tissue remodelling is immature and may be susceptible to degradation.^64^ Two large cohort studies have identified tenascin immunohistochemistry as the most sensitive method for detecting collagen in CG.^34^, ^38^ Among the other components of the ECM, laminin has been reported to be negative in CG cases.^8^ The rest of the families of procollagens and matrix‐metalloproteinase have not been tested in CG. Further characterization of the components of the collagen band and associated matrix proteins may yield important insights in future studies.

### Inflammatory Changes in the Gastric Mucosa

**Figure 2:**
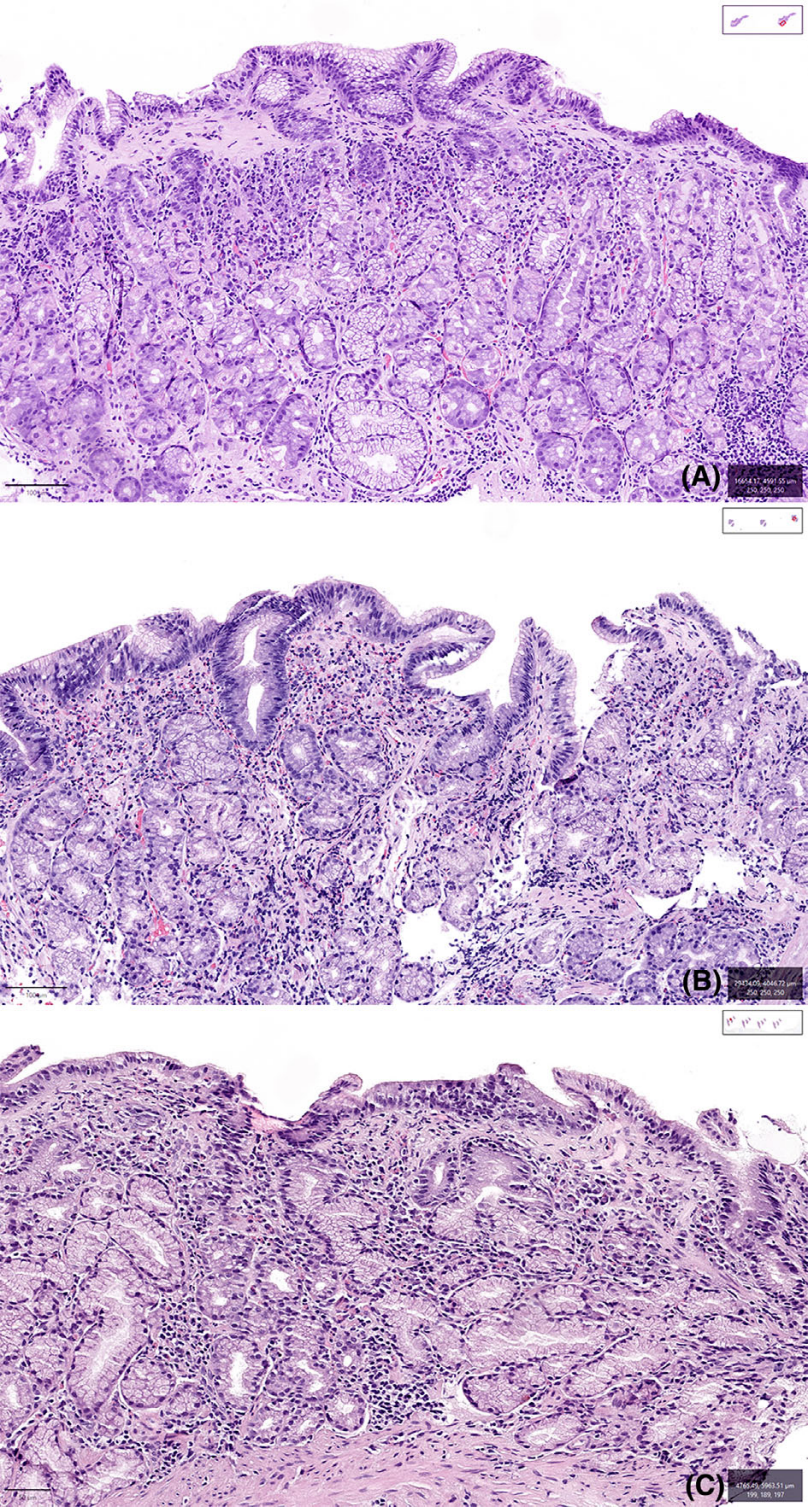
Collagenous gastritis is characterized by thickened subepithelial collagen, and the inflammatory component can be classified into three distinct histological patterns: atrophic, lymphocytic gastritis‐like and eosinophil‐rich. (**A**) (top, H&E, 100×) shows the atrophic pattern, characterized by prominent glandular atrophy, particularly parietal cell loss in the gastric body. Pseudopyloric gland metaplasia may be present, but intestinal metaplasia or enterochromaffin‐like cell hyperplasia is typically absent, excluding autoimmune gastritis. (**B**) (middle, H&E, 100×) shows the eosinophil‐rich pattern, which demonstrates a predominance of eosinophils, occasionally with abundant eosinophils entrapped within the thickened collagen table. (**C**) (bottom, H&E, 200×) shows the lymphocytic gastritis‐like pattern, with an increase in intraepithelial lymphocytes, which may or may not exceed the threshold of 25 lymphocytes per 100 epithelial cells used in the diagnosis of lymphocytic gastritis.

The characterization of inflammation in CG in the literature remains largely descriptive, with limited recognition of histologic patterns. This is primarily due to the rarity of the disease and the predominance of single case reports or small case series in the literature. One of the few studies offering broader insight is the comprehensive pathology series by Arnason *et al*. which identified three distinct histologic patterns in CG: an atrophic pattern, a lymphocytic gastritis‐like pattern and an eosinophil‐rich pattern, with representative examples shown in Figure 2. For the purpose of this review, histologic descriptions from the published literature—where available and sufficiently detailed—were re‐examined, categorized according to these patterns and are summarized below. While the atrophic and lymphocytic gastritis patterns can often be reasonably inferred from available descriptions, the eosinophil‐rich pattern is likely underrecognized and underreported. This may be due to an overall abundance of eosinophils in the gastrointestinal tract and the absence of clear diagnostic thresholds for reporting eosinophils, especially when eosinophils, though prominent, are present as a secondary finding. Additionally, a focused literature review did not identify relevant studies addressing this specific topic, underscoring the limitations of the current evidence base in facilitating further differentiation of such cases.

Based on the review of literature, the atrophic pattern characterized by glandular atrophy in association with chronic gastritis is the most commonly observed pattern in CG. Accompanying neutrophils and intraepithelial lymphocytes have been observed in a small number of cases.^11^, ^35^, ^38^, ^62^, ^65^, ^66^ Most cases of atrophic pattern CG demonstrate isolated gastric involvement. Fewer than half of the patients in this group demonstrate clinical remission; notably, histologic remission is even rarer and has been reported in only a limited number of cases.^56^, ^66^, ^67^

In approximately two‐thirds of atrophic pattern CG cases, atrophy is confined to the gastric corpus^1^, ^6^, ^35^, ^38^, ^43^, ^44^, ^56^, ^66^, ^68^ and is typically characterized by parietal cell loss, with or without pseudopyloric metaplasia as shown in Figure 2A. Notably, intestinal metaplasia and neuroendocrine cell hyperplasia are absent in the majority of cases. An absence of gastrin expression can help identify this pattern in cases where the site of the biopsy is not clear.^38^ While these histologic features may raise suspicion for autoimmune gastritis (AIG), the presence of a subepithelial collagen band—a hallmark of CG—and the lack of other metaplastic changes help distinguish it from AIG. Furthermore, autoimmune serologic testing has generally been negative in reported cases.^40^

Rare instances have documented the progression of atrophic pattern CG to intestinal metaplasia and neuroendocrine cell hyperplasia. One such case, initially reported by Collier *et al*. and later by Winslow *et al*. describes the clinicopathologic evolution of CG in an individual over 12 years, involving evaluation of 109 biopsies from 19 different endoscopic procedures. These reports illustrate the progression from a corpus‐predominant atrophic CG pattern to the development of neuroendocrine cell hyperplasia, mild to moderate glandular atrophy with intestinal metaplasia and reactive epithelial changes indeterminate for dysplasia—findings that raised concern for potential adenocarcinoma.^1^, ^69^

In about one‐third of atrophic CG cases, these changes diffusely involve both the antrum and corpus, again mostly without intestinal metaplasia. Approximately half of these cases with diffuse gastric atrophy also exhibit collagenous involvement of other gastrointestinal sites, such as collagenous colitis or collagenous sprue.^6^, ^20^, ^35^, ^56^, ^65^, ^70^, ^71^, ^72^ A subset of diffuse atrophic patterns also has associated or preceding *H. pylori* infection.^20^, ^56^, ^71^

The lymphocytic gastritis (LG)‐like pattern is more commonly associated with collagenosis at other sites within the gastrointestinal tract. A less frequent but notable association in this group is celiac disease or atypical celiac disease.^15^, ^18^, ^38^, ^39^ The inflammatory infiltrate in this group may also contain neutrophils and prominent eosinophils, in addition to intraepithelial lymphocytes (IELs), and the disease often shows an antral‐predominant distribution.^15^, ^18^, ^39^ Clinical resolution following a gluten‐free diet has been reported in select cases.^34^, ^39^, ^52^

Following the exclusion of atrophic and lymphocytic gastritis‐like patterns, the remaining cases are described as non‐descript, more commonly diffuse, chronic active or inactive patterns of gastritis. Prominent eosinophils are reported in only a subset of this group.^5^, ^9^, ^22^, ^31^, ^32^, ^36^, ^48^, ^49^, ^52^, ^57^, ^58^, ^73^, ^74^, ^75^ Patients in this group show an almost equal association with atypical celiac disease, collagenosis at other gastrointestinal sites, *H. pylori* gastritis or isolated gastric disease.^9^, ^15^, ^21^, ^31^, ^32^, ^34^, ^48^, ^52^, ^57^, ^58^, ^73^, ^74^, ^75^ The reported rate of clinical remission is relatively higher in this subgroup.^5^, ^9^, ^22^, ^36^, ^48^, ^49^, ^52^, ^57^, ^74^, ^75^

As noted above, classifying gastric inflammation by histologic patterns not only allows for more reproducible characterization of CG but also provides insight into potential differential diagnoses, associated clinical and histologic phenotypes and disease outcomes to some extent. As such, we suggest that pathology reports clearly include these features, especially considering the knowledge gap due to the rarity of this disease. Suggested components for inclusion in pathology reports are listed below and illustrated in the graphical abstract.

### Suggested Components in the Pathology Report of CG

1. Collagen band:
  - Thickness and location: Include measurements and anatomical location of the collagen band
  - Associated features: Presence of ‘trapillaries’ and/or surface epithelial detachment
  - Special stains: Results of Masson trichrome (or equivalent collagen stain), Congo red and additional stains such as tenascin, collagen sub‐typing or ultrastructural studies, if performed
2. Gastritis:
  - Pattern: Atrophic, lymphocytic gastritis‐like, eosinophil‐rich or nonspecific pattern (Figure 2)
    - Atrophic changes:
      - Presence or absence of corpus and/or antral glandular atrophy
      - Presence or absence of neuroendocrine cell hyperplasia, especially in corpus biopsies
      - Presence or absence of intestinal metaplasia
  - Location: Corpus and/or antrum/pylorus
  - Inflammatory infiltrate components: Neutrophils, eosinophils, intraepithelial lymphocytes
  - *Helicobacter* pylori organisms: Present or absent
3. Additional notes:
  - Clinical and histologic associations: When diagnosing collagenous gastritis (CG), the pathology report should comment on potential associated conditions. CG may occur as an isolated gastric finding, but it can also be seen in association with collagenous disease in other parts of the gastrointestinal tract, celiac disease (including atypical forms), *H. pylori* gastritis and, more rarely, drug‐induced aetiologies (e.g. olmesartan). Addressing these underlying conditions has been associated with clinical improvement in a subset of cases.
  - Recommendations for follow‐up: Given the chronic and often protracted course of CG, consider recommending gastric mapping biopsies in follow‐up to monitor histologic progression or response to treatment.

**Figure 3:**
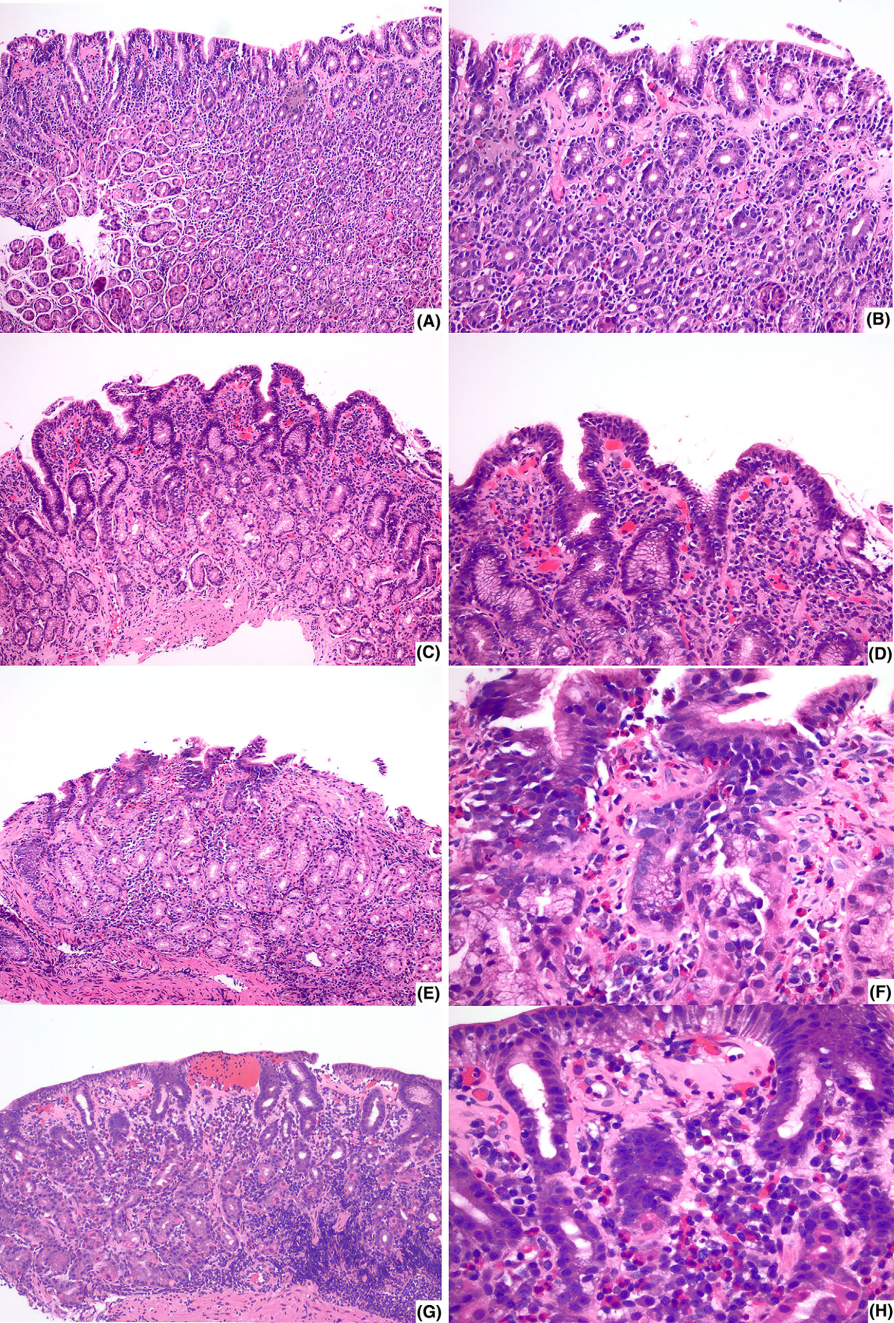
Histologic spectrum of changes in collagenous gastritis, illustrating variability in intraepithelial lymphocytosis and eosinophilia. Top panel (**A–D**): H&E‐stained gastric biopsies demonstrating a spectrum from increased intraepithelial lymphocytes in surface and pit epithelium (**A** and **B**, H&E, 100× and 200×) to only focal prominence predominantly in the surface epithelium (**C** and **D**, H&E, 100× and 200×). Bottom panel (**E**–**H**): H&E‐stained sections highlighting variable eosinophilic inflammation, ranging from a predominantly eosinophilic infiltrate with degranulation (**E** and **F**, H&E, 100× and 400×) to focal prominence of eosinophils admixed with dense lymphoplasmacytic infiltrate (**G** and **H**, H&E, 100× and 400×). This panel underscores the histologic variability seen in collagenous gastritis and highlights the need for standardized threshold‐based quantification of intraepithelial lymphocytes and eosinophils, in addition to pattern recognition, to ensure consistent diagnostic interpretation.

Interpretation of histologic patterns requires careful consideration of sampling variability, as patients frequently undergo biopsies from multiple anatomical sites and timepoints, and involvement by a given pattern may be patchy. This heterogeneity may result in the identification of multiple patterns within a single case. Moreover, the degree and distribution of intraepithelial lymphocytes and density and proportion of eosinophilic infiltrate can vary significantly, as illustrated in Figure 3. These factors may affect interobserver reproducibility and contribute to diagnostic inconsistency. To address such challenges, future studies are needed to develop and validate stringent, reproducible criteria for pattern classification, such as using not only a defined threshold for intraepithelial lymphocyte (IEL) density but also a requisite proportion of involved biopsies to categorize a case as showing an LG‐like pattern. Adoption of such standardized criteria would facilitate more accurate correlations with disease associations and improve prognostic stratification.

## Unanswered Questions: Theoretical Insights and Genetic Evidence in Pathogenesis of Collagenous Gastritis

The pathogenesis of CG remains unclear, with several hypotheses proposed to explain the origin of the subepithelial collagen band, a hallmark of the disease. These include an inflammatory aetiology, local abnormalities of the pericryptal collagen sheath and immune‐mediated injury.^12^, ^20^, ^25^, ^34^, ^39^, ^40^, ^43^, ^49^, ^76^ One theory posits that the initial trigger for both atrophic gastritis and CG is shared; however, once initiated, collagen deposition in CG progresses independently of the ongoing mucosal inflammatory activity.^56^, ^65^

Activated fibroblasts have been identified in the gastrointestinal mucosa of affected individuals, which may account for the simultaneous collagen deposition in both colon and stomach in cases of synchronous CG and CC.^49^ A potential decrease in collagen turnover has also been suggested, although the exact stimulus that activates the subepithelial fibroblasts is still unclear.^1^ Additional proposed hypothesis involves leakage of plasma proteins and fibrinogen into the lamina propria, potentially contributing to the formation of the collagen band.^77^

The role of infectious agents in CG pathogenesis remains debated. Some reports argue against a significant role for bacterial infection, while others suggest that *H. pylori* infection could act as a trigger through persistent mucosal inflammation.^8^, ^78^ In a few cases, treatment of *H. pylori* has been associated with partial regression of CG, further supporting a potential link.^20^

Given the frequent co‐occurrence of autoimmune diseases in patients with CG, an autoimmune‐mediated mechanism has been considered. In a cohort of 15 patients, 47% reported a family history of autoimmune disorders, suggesting a possible genetic predisposition.^33^ However, a clear mechanistic link between autoimmunity and CG has not yet been established.

Another proposed hypothesis involves the role of eosinophils. The frequent presence of eosinophilic infiltrates has raised the possibility that eosinophilic degranulation may contribute to fibrotic remodelling. This could be initiated by unidentified intraluminal antigen, whose continued presence in the diet may explain the refractoriness of disease to pharmacologic treatment.^69^ The role of eotaxin, a chemokine involved in the selective recruitment and activation of eosinophils, and a key player in eosinophil‐driven inflammation and tissue remodelling has been investigated in collagenous gastritis. In one study, eotaxin immunohistochemical staining did not show a significant increase in cases of collagenous gastritis, suggesting a limited or unclear role in its pathogenesis. However, as noted by the authors, this conclusion may be confounded by the low sensitivity of immunohistochemistry. More sensitive techniques, such as quantitative real‐time PCR, would be necessary to more definitively evaluate eotaxin's involvement.^38^ IgG4‐related disease is now recognized as a systemic condition that can affect virtually any organ or tissue, often presenting with nonspecific symptoms. In a reported case of collagenous gastritis with infiltration by IgG4‐positive plasma cells, immunosuppressive therapy led to clinical improvement, suggesting that IgG4 may also be involved in the pathogenesis of CG in some cases.^42^ However, a study by Arnason *et al*. identified only one patient with elevated IgG4‐positive plasma cells in the biopsies meeting the criteria for IgG4‐related disease. Given the rarity of this finding and the absence of systemic involvement in any of the patients in their study, they concluded IgG4 is unlikely to play a significant role in the pathogenesis of collagenous gastritis.^38^ Recent studies have begun to explore the molecular mechanisms underlying CG, though data remain limited. In 2022, Curci *et al*. conducted the first proteomic analysis using SOMAscan technology on serum samples from nine paediatric CG patients.^79^ They identified 63 significantly dysregulated proteins from 1,305 biologically relevant proteins covered in the panel. These proteins are linked to enhanced inflammatory responses, immune cell migration and impaired vascular function; of these, 17 are associated with fibrosis. Notably, a two‐fold reduction in EGF levels and increased FGF19 levels were seen in CG patients compared with non‐CG or non‐gastritis controls, suggesting their potential as biomarkers for CG. The authors also concluded a role of EGF in the pathophysiology of collagenous gastritis. STRING analysis performed identified EGF as a central focus hub in pathways related to extracellular matrix collagen turnover and fibrillogenesis, vascularization and epithelial remodelling. Notably, EGF under several pathophysiological conditions promotes collagen breakdown due to its enhancement of collagenase expression. At the same time, it reduces collagen gene transcription, suppressing its synthesis; thus, its reduced expression in CG may contribute to pathological collagen accumulation. In 2023, *Liu et al*. performed the first gene expression profiling of CG using Nanostring nCounter assay with a coverage of 594 inflammation‐related genes and 184 immunology‐related genes in 10 newly diagnosed patients.^80^ The results revealed a distinct upregulation of both Th1 (IFNγ, TNF‐α, IL‐2, IL‐10, IL‐12A, IL‐12B and IL‐18) and Th2 (IL‐3, IL‐4, IL‐5, IL‐6 and IL‐13) cytokines encoding genes in CG, contrasting with the Th1‐only profile seen in CC. Th2 cytokines (IL‐4, IL‐5, IL‐13) drive mast cell and eosinophil activation and support antibody responses to parasites and allergens, while counterbalancing Th1‐mediated inflammation. The authors concluded that the distinct Th2 unique signature potentially recruits eosinophils by IL‐5, which may contribute to fibrosis through TGF‐β1 production, promoting collagen deposition and tissue remodelling. Upregulation of genes related to collagen metabolism, *matrix metallopeptidase 3*, *matrix metallopeptidase 9* and *leukocyte immunoglobulin‐like receptor sub‐families A and B* was also seen. The authors also highlight the link of this distinctive Th2 signature and a potential role for environmental allergens as triggers or exacerbating factors in CG, highlighting the possible benefit of dietary interventions, particularly hypoallergenic or gluten‐free diets. It also supports the therapeutic potential of combined anti‐inflammatory, antiallergic and Th2‐targeted treatments, such as interleukin receptor modulators (e.g. mepolizumab). Furthermore, it may explain the reported clinical and histologic improvements with topical budesonide, likely attributable to its dual anti‐inflammatory and allergy‐modulating effects.

Despite growing insights into the immunologic and proteomic features of CG, the genetic landscape of the disease remains largely unexplored. To date, the potential contribution of inherited defects in collagen synthesis, modification or degradation to the pathogenesis of CG has not been systematically explored. This represents an area of interest given that some genetic defects of collagen genes can be challenging to detect and may not present with overt features of connective tissue disorders.^81^, ^82^, ^83^ Considering that subepithelial collagen deposition is a defining histologic feature of CG, investigating potential underlying genetic factors could offer valuable insights. Adding to this complexity, a recent study reported two unrelated patients with a chronic atrophic gastritis pattern mimicking CG, who were found to carry pathogenic variants in telomeropathic genes (*POT1* and *DCLRE1B*). Both cases exhibited severe loss of stem/progenitor cells within the gastric mucosa, suggesting that impaired regenerative capacity may drive mucosal atrophy in a subset of patients.^84^ These findings suggest that CG may not represent a single disease entity, but rather a shared histologic phenotype that can result from a range of explored and as‐yet unexplored aetiologies, including allergen‐driven inflammation, genetic defects in collagen biology, impaired epithelial homeostasis, immune dysregulation or other yet unconsidered triggers.

## Treatment

The standard treatment and evidence‐based guidelines for management of CG have not yet been established, due to the poor understanding of disease aetiology, as well as the small number of patients and variable natural course, making clinical trials extremely challenging. Current treatment includes symptomatic treatment, including iron supplement, lansoprazole and vitamin B12 replacement therapy. Proton pump inhibitors are also often used. Other exploratory treatments include a gluten‐free diet, even in patients without duodenal involvement.^50^, ^74^ Steroids, including budesonide and prednisone, as well as sulfasalazine, have also been used, resulting in variable treatment responses.^8^, ^27^ Significant symptomatic and histologic improvement following vedolizumab infusion has been reported, including decreased inflammatory infiltrate and re‐appearance of healthy foveolar cells; however, the collagen bands remained unchanged.^67^

While this review provides a comprehensive update on collagenous gastritis, readers may also find the section on collagenous gastritis in the *Inflammatory Disorders of the Stomach* by Won‐Tak Choi *et al*., included in *Morson and Dawson's Gastrointestinal Pathology* textbook, to be a concise and valuable resource for additional context.^85^ Limitations of this review include the inclusion of English‐language‐only publications that explicitly listed collagenous gastritis as a keyword and provided discernible clinical, endoscopic and histologic data. Given that CG can present with several histologic patterns of injury, relevant cases may have been missed, particularly within broader cohort studies or those focusing on drug‐mediated gastrointestinal injury where CG was not specifically identified. Despite these limitations, the findings underscore the need for future studies, particularly those incorporating genomic sequencing and molecular profiling, to refine histologic subtypes, identify potential genetic subtypes and clarify their clinical implications.

## Author contributions

N.S.—Study conceptualization; J.L. and N.S.—Literature review; J.L., K.T. and N.S.—Manuscript writing; K.T. and N.S.—Figure preparation; J.L. and N.S.—Manuscript revision. All authors read and approved the final manuscript.

## Funding information

None.

## Conflict of interests

None.

## Data Availability

The data that support the findings of this study are available from the corresponding author upon reasonable request.
